# ER Stress and Unfolded Protein Response in Leukemia: Friend, Foe, or Both?

**DOI:** 10.3390/biom11020199

**Published:** 2021-01-30

**Authors:** Kelly Féral, Manon Jaud, Céline Philippe, Doriana Di Bella, Stéphane Pyronnet, Kevin Rouault-Pierre, Laurent Mazzolini, Christian Touriol

**Affiliations:** 1 Inserm UMR1037-Cancer Research Center of Toulouse, 2 avenue Hubert Curien, Oncopole entrée C, CS 53717, 31037 Toulouse, France; kelly.feral@inserm.fr (K.F.); manon.jaud@inserm.fr (M.J.); stephane.pyronnet@inserm.fr (S.P.); 2 Université Toulouse III Paul-Sabatier, F-31000 Toulouse, France; 3 Barts Cancer Institute, Queen Mary University of London, London E1 4NS, UK; c.philippe@qmul.ac.uk (C.P.); doriana.dibella@qmul.ac.uk (D.D.B.); k.rouault-pierre@qmul.ac.uk (K.R.-P.); 4 CNRS ERL5294, CRCT, F-31037 Toulouse, France

**Keywords:** endoplasmic reticulum stress, unfolded protein response (UPR), leukemia, AML, CLL, ALL, CML

## Abstract

The unfolded protein response (UPR) is an evolutionarily conserved adaptive signaling pathway triggered by a stress of the endoplasmic reticulum (ER) lumen compartment, which is initiated by the accumulation of unfolded proteins. This response, mediated by three sensors-Inositol Requiring Enzyme 1 (IRE1), Activating Transcription Factor 6 (ATF6), and Protein Kinase RNA-Like Endoplasmic Reticulum Kinase (PERK)—allows restoring protein homeostasis and maintaining cell survival. UPR represents a major cytoprotective signaling network for cancer cells, which frequently experience disturbed proteostasis owing to their rapid proliferation in an usually unfavorable microenvironment. Increased basal UPR also participates in the resistance of tumor cells against chemotherapy. UPR activation also occurs during hematopoiesis, and growing evidence supports the critical cytoprotective role played by ER stress in the emergence and proliferation of leukemic cells. In case of severe or prolonged stress, pro-survival UPR may however evolve into a cell death program called terminal UPR. Interestingly, a large number of studies have revealed that the induction of proapoptotic UPR can also strongly contribute to the sensitization of leukemic cells to chemotherapy. Here, we review the current knowledge on the consequences of the deregulation of UPR signaling in leukemias and their implications for the treatment of these diseases.

## 1. Introduction

About one-third of human genes encode secreted or transmembrane proteins as well as proteins resident of the endoplasmic reticulum, the Golgi apparatus, and lysosomes. Most of these proteins are targeted to the ER. The endoplasmic reticulum is a complex network of membrane-enclosed tubules and vesicles, extending from the nuclear membrane throughout the cytoplasm. ER is the largest organelle of most eukaryotic cells, as its membrane may account for at least 50% of all cell membranes and even more for specialized secretory cell. Its total area is 10–30 times that of the plasma membrane. ER constitutes the first compartment of the secretory pathway in which secreted and transmembrane proteins are folded and post-translationally modified [1].

ER is also the most important compartment for intracellular calcium ions (Ca2+) storage, which is necessary for the physiological activities of the ER, allowing the maintenance of the oxidation–reduction potential [2,3]. In its lumen, a set of specialized proteins like chaperones, foldases, glycosylating enzymes, oxidoreductases, and cofactors ensures the correct folding of newly synthesized proteins. By interacting with the exposed hydrophobic segments present on the newly synthesized proteins or on misfolded proteins, the chaperones (BiP/GRP78, calnexin, GRP94, etc.) act both to complete the folding process and to correct folding errors [4]. After passing the protein quality control checkpoints in the ER, correctly folded proteins traffic via the Golgi to other organelles and/or to the plasma membrane. Despite this optimized environment in the ER luminal domain, the success rate for accurate folding is variable. In case of unsuccessful folding, proteins are released in the cytosol where they become ubiquitinated and targeted to degradation by the proteasome. This rigorous quality control system has been named ERAD for Endoplasmic Reticulum-Associated Degradation [5].

In addition, to cope with the perturbations caused by unfolded or misfolded proteins, cells set off an adaptive response called the unfolded protein response (UPR), which aims to restore normal ER functioning [6,7,8,9]. This is achieved by (i) lowering the biosynthesis of proteins to reduce accumulation of misfolded proteins in the ER; (ii) increasing the biosynthesis of chaperone proteins; (iii) increasing ER size through membrane synthesis, (i), and (ii) resulting in a boost of ER folding capabilities; and finally (iv) increasing the biosynthesis of ER-associated degradation proteins thus improving the cell’s ability to eliminate misfolded proteins. Consequently, “adaptive UPR” limits cell damages and allows cell recovery and survival to a new stressful environment. However, if stress overcomes cell recovery capacities UPR can switch from an adaptive to a “terminal UPR” program triggering cell death [10,11,12].

Perturbations in the ER stress response such as either chronic ER stress or defects in UPR signaling, have been associated with a number of pathologies: diabetes, atherosclerosis, inflammation, stroke, pulmonary fibrosis, several eye diseases, neurodegenerative disorders (including amyotrophic lateral sclerosis, Alzheimer’s, Parkinson’s or Huntington’s diseases), and, of course, cancer [13,14,15,16]. The common feature among these seemingly different diseases is a cellular dysfunctioning leading to an accumulation of misfolded proteins in the ER.

With respect to cancer, the role of ER stress response/UPR signaling pathways was mainly studied in primary solid tumors in which a very unfavorable microenvironment mainly originating from inadequate vascularization and characterized by nutrient (e.g., amino acids, glucose) deprivation, hypoxia, acidosis leads to the activation of ER stress in the highly proliferative and metabolically active cancer cells [17,18,19,20,21]. However, in recent years our current knowledge on the essential functions played by the UPR in leukemia has also significantly improved.

In this review, after introducing the Unfolded Protein Response, we will summarize current findings on the involvement of ER stress in the progression of leukemia, and discuss the potential therapeutic effects of UPR activation or repression in these pathologies.

## 2. The Unfolded Protein Response

**Figure 1:**
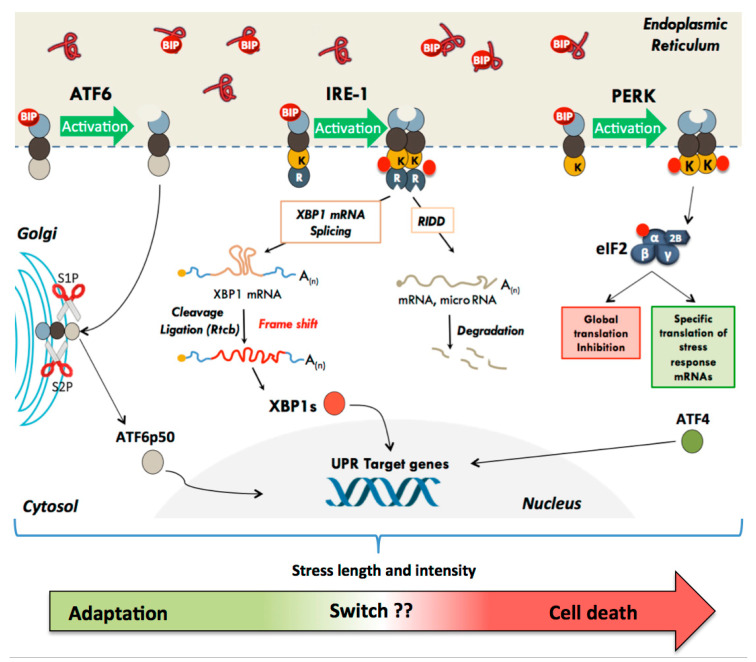
The different UPR effectors and their modes of action. In the basal state, the three UPR effector transmembrane proteins (PERK, ATF6, and IRE-1) are maintained inactive through their interaction with the protein chaperone BiP. The accumulation of misfolded proteins in the ER lumen results in dissociation of BiP and activation of UPR. (1) PERK dimerizes and phosphorylates the eIF2α subunit, leading to a global inhibition of translation initiation. Specific mRNA subsets, containing cis-acting elements in their 5′UTR, such as uORF and IRES, escape translational inhibition triggered by eIF2 phosphorylation. (2) IRE-1 initiates an unconventional splicing of XBP-1 mRNA. IRE1α cleaves Xbp1u mRNA within two stem-loop structures, leading to excision of 26 nucleotides. Subsequent ligation of the Xbp1 mRNA by the tRNA ligase RTCB results in a frame shift and allows the translation of the active transcription factor XBP1s, which is imported into the nucleus and activates the expression of target genes. IRE1α mediates also the degradation of some RNAs (this mechanism has been called RIDD for Regulated Ire1-Dependent Decay). (3) BIP dissociation from ATF6 exposes its Golgi Localization Signal. ATF6 is translocated to the Golgi apparatus where proteolysis releases its transcription factor amino-terminal domain, which is imported into the nucleus and activates the expression of target genes. The UPR has a primary function in adaptive response in order to restore homeostasis and promote cell survival, but depending on the duration and intensity of the stress, a switch can induce cell death to get rid of the damaged cells.

In mammals, UPR is triggered by activation of three ER transmembrane sensors: PERK (PKR-like ER-associated protein kinase), ATF6 (Activating Transcription Factor-6), and IRE1 (inositol-requiring enzyme-1) [6,10,22,23]. The luminal part of these proteins integrates the information coming from the ER lumen, whereas their cytosolic part interacts with their effectors and mediates the signaling cascades (Figure 1). In the absence of stress, the ER resident protein chaperone BiP also known as GRP78 (Glucose-regulated protein 78kDa) or HSPA5 (Heat Shock Protein Family A (Hsp70) Member 5) binds to the luminal domain of the three effectors and keep them in an inactive state. Upon accumulation of unfolded proteins in the ER lumen, BiP will act as a protein chaperone, interact with exposed hydrophobic segments of misfolded proteins, and thus be released from ATF6, IRE1, and PERK, leading to their activation [24,25]. In addition to BiP release, an activation of IRE1 by oligomerization induced by direct binding of unfolded proteins has also been reported, both in yeast [26] and mammalian cells [27]. Therefore, the relative ratios of three proteins complexes inside the endoplasmic reticulum, namely, those created by interaction between BiP and either unfolded proteins or UPR sensors, as well as those formed by direct interaction between unfolded proteins and the UPR sensors themselves, could contribute to a very precise and dynamic regulation of the UPR [28].

As previously stated, the primary goal of the activated signaling cascades is to reestablish ER homeostasis by a two-step process: in a first stage, through the reduction of overall protein synthesis and the degradation of misfolded proteins, and in a second stage through the activation of cellular functions crucial for cell survival [6,10,22].

However, in the absence of protein homeostasis restoration, the adaptive UPR will switch to terminal UPR, which ultimately results in cell death. Cell fate is largely influenced by the intensity and duration of the stress. A long or intense stress leads to the activation of this terminal UPR [10,18]. The regulatory networks, which determine the transition from adaptive to terminal UPR, are complex and not fully understood. Regardless, the molecular events that will direct the cell towards either adaptive or terminal UPR involve to some extent each of the PERK, IRE1, and ATF6 signaling cascades. The contribution of each pathway to the execution of the adaptive or terminal UPR may be variable depending on the type of cell and on the nature and extent of damage experienced by the cell. The different UPR signaling cascades are described below.

### 2.1. The Translational Pathway: Activation of the PERK Kinase

Among the three key proteins involved in UPR, PERK (encoded by the EIF2AK3 gene for eukaryotic translation initiation factor 2-alpha kinase 3) is the first to be activated by autophosphorylation. The dissociation of BiP from its luminal domain causes dimerization or oligomerization and trans-autophosphorylation of PERK (threonine 981), thus activating the cytosolic serine/threonine kinase domain (Figure 1). The main substrate of PERK is the alpha subunit of the translation initiation factor eIF2 (eukaryotic Initiation Factor 2) [29,30,31,32]. The eIF2 factor, which possesses three subunits-α, β, and γ-links the initiator methionine tRNA (tRNA-Met) to the small ribosomal subunit. The regulatory α subunit contains a serine (ser51) strictly conserved in eukaryotes. By phosphorylating ser51, PERK induces a global inhibition of cap-dependent translation initiation and therefore overall protein synthesis in order to temporary reduce unfolded protein load, until favorable conditions return [33]. Mechanistically, the phosphorylation of the eIF2α ser51 increases the affinity of eIF2 for its own eIF2B guanine nucleotide exchange factor (GEF), which recycles the inactive form of eIF2-GDP into its active form eIF2-GTP. This strong interaction induces sequestration of the eIF2 factor by eIF2B, causing a blockade of active translation pre-initiation complex formation and thus inhibition of translation initiation [34]. This translation inhibition prevents further protein loading in the ER, reduces cell overall metabolism and saves energy to repair the damage caused by the stress [34].

In parallel to cap-dependent translation arrest, translation of specific messenger RNAs exhibiting particular features in their 5′ untranslated region is selectively induced [35]. This is the case of the Activating Transcription Factor 4 (ATF4) mRNA which contains several upstream open frames (uORFs) in its 5′ untranslated region, preventing translation of the main open reading frame (ORF) in normal conditions. Under stress conditions however, low levels of active eIF2α allow the ribosomes to reach the main ATF4 ORF and efficiently initiate translation of this transcription factor, which in turn activates the expression of chaperones and of genes involved in amino acid metabolism and resistance to oxidative stress [36,37]. Interestingly, some mRNA whose translation depends on the presence of internal ribosome entry sites (IRESs) in their 5′ untranslated region [35] and coding for stress response proteins are also activated when eIF2α is phosphorylated (Figure 1) [38,39,40,41].

The dephosphorylation of eIF2α is necessary to restore a normal protein synthesis level after stress. This reset to the basal state is achieved by two phosphatases, composed of a single catalytic subunit PP1 (Protein Phosphatase 1) and one of the two regulatory subunits GADD34 (Growth And DNA-Damage inducible protein 34) or CReP (Constitutive Repressor of eIF2α phosphorylation) [42]. In contrast to CReP, which is constitutively expressed, the expression of GADD34 is only induced in response to stress as a negative feedback loop [43]. Indeed, transcription of GADD34 is activated by ATF4 and its translation is, as for ATF4 itself, regulated by a uORF mechanism ensuring proper GADD34 expression despite eIF2α phosphorylation [44].

Under chronic stress, sustained activation of PERK and thus prolonged expression of ATF4 induce apoptosis by activating CHOP transcription (C/EBP Homologous protein, also known as GADD153-Growth And DNA-Damage inducible protein 153 or DDIT3-DNA-Damage Inducible Transcript 3) [45]. This transcription factor, a member of the CCAAT/enhancer-binding protein (C/EBP) family, plays a central, multifunctional role in the UPR-induced apoptotic process [46]. CHOP can alone or cooperatively with other transcriptional factors function either as a transcriptional activator or repressor. It acts mainly by modulating the expression of various members of the BCL-2 protein family playing either pro-(Bim) or antiapoptotic (Bcl-2, BCL-XL and MCL-1) functions [47]. CHOP can however also induce cell death by many additional, non-exclusive, pathways such as restoration of protein synthesis (via GADD34 activation) which leads to increased proteins load detrimental to the cell (“proteotoxicity”) and by increased ROS production (through upregulation of the ER reductase ERO1α) [48].

It is interesting to note that eIF2α is not the only PERK substrate. Indeed, the transcription factor Nrf2 (Nuclear Factor (erythroid derived 2)-like2), which is involved in the response to oxidative stress, is normally maintained in the cytoplasm by association with Keap1. Under stress conditions, PERK phosphorylates Nrf2. This causes a dissociation of the Nrf2/Keap1 complex and allows the import of Nrf2 to nuclear compartment [49]. Nrf2 then bind to ARE sequences (Antioxidant response element) on the promoter of its target genes such as GCLC (Glutamate Cysteine Ligase Catalytic Subunit), HO-1 (Heme oxygenase 1) or NQO1 (NADPH dehydrogenase quinone 1) [50]. Thus, the activation of Nrf2 by PERK helps in maintaining the redox status of the cell subjected to ER stress.

### 2.2. The Transcriptional Pathway: Activation of ATF6α and IRE1α

In mammals, the transcriptional response to ER stress involves two families of transmembrane proteins: the IRE1 and ATF6 proteins (Figure 1).

The ATF6α (activating transcription factor 6 α) transcription factor is a type II transmembrane protein characterized by a C-terminal luminal domain, sensitive to misfolded proteins, and an N-terminal cytosolic portion containing a leucine zipper DNA binding domain (bZIP) and a transcriptional activation domain. In mammals, two ATF6 proteins, ATF6α and ATF6β, are produced form independent genes. Whereas both proteins are ubiquitously expressed, only ATF6α has proven to be an effective transcriptional activator and its it is currently accepted that only ATF6α plays a major role in the ATF6-dependent transduction of UPR signaling [51]. The amount and mode of contribution of ATF6β to the unfolded protein response remain poorly understood and need to be further investigated [52]. During ER stress, Bip dissociation from the ATF6α protein allows the exposure of two Golgi localization signals, and migration of ATF6α from ER to the Golgi apparatus where it undergoes 2 sequential cleavages by the proteins S1P and S2P (Site-1 and Site-2 Proteases) (Figure 1) [25,53]. These cleavages generate a transcriptionally active N-terminal short-lived fragment of 50 kDa called ATF6p50 which translocates into the nucleus to activate the transcription of chaperone and foldase proteins such as BiP, calreticulin, calnexin, and protein disulfide isomerases. ATF6p50 also activates the transcription of enzymes such as the calcium pump SERCA (sarco/endoplasmic reticulum Ca2+-ATPase). This ER ATPAse transports calcium ions from the cytosol into the ER and plays a major role in the maintenance of calcium homeostasis which controls many essential cellular processes [54]. ATF6p50 also promotes the expression of different genes involved in lipid biosynthesis, thus participating to the expansion of the endoplasmic reticulum [55]. It also upregulates XBP1 (X-box binding protein 1), a transcription factor which acts immediately downstream of the third UPR sensor IRE1 (see below). Moreover, ATF6α can also form heterodimers with XBP1 and upregulate genes involved in the ERAD pathway like EDEM (ER Degradation Enhancing Alpha-Mannosidase Like Protein 1) or HERPUD1 (Homocysteine Inducible ER Protein With Ubiquitin Like Domain 1). ATF6α gene invalidation induces increased sensitivity to ER stress probably due to impaired induction of chaperone proteins such as BiP or GRP94 (Glucose-regulated protein 94 kDa) [56,57]. However, ATF6α can also activate the expression of the proapoptotic factor CHOP [58,59], and a very recent work suggested that ATF6α could play an important role in the decision from adaptive to terminal UPR by modulating early and late CHOP expression kinetics [60]. Therefore, the role played by ATF6α on cell survival or death appears complex. In addition, the ATF6α transactivator domain (more precisely the first N-terminal 93 amino acids) has been shown to be responsible for its own degradation by the proteasome [61]. As a result, ATF6α appears as a powerful transcriptional activator, but with a transient effect. This may contribute to finely tune the UPR machinery.

The third UPR sensor is IRE1 (Inositol-requiring protein 1 also known as ERN1 for Endoplasmic reticulum-to-nucleus signaling 1), a 110 kDa protein initially identified in yeast where it is the only ER stress sensor. In mammals this protein is expressed as two isoforms: IRE1α, which is ubiquitously expressed, and IRE1β expressed only in the epithelial cells of the digestive system [62,63,64,65,66]. IRE1α possesses a luminal structure and an activation mode similar to that of PERK. However, in addition to a Ser/Thr kinase enzymatic activity, the IRE1α cytosolic domain also retains an atypical endoribonuclease (RNAse) activity, which becomes functional after IRE1α homodimerization under stress conditions [67]. This dimerization is essential for endoribonuclease activation, which is also dependent on IRE1α phosphorylation status [68]. The IRE1α RNAse domain catalyzes the excision of a 26-nucleotide sequence within the Xbp1 (X-box binding protein1) mRNA by an unconventional cytoplasmic splicing mechanism independent of the spliceosome (Figure 1) [69]. This cleavage, followed by a ligation step mediated by the RTCB tRNA ligase [70], generates a frame shift in the open reading frame, which leads to the expression of XBP1s (XBP1 spliced), a transcription factor belonging to the ATF/CREB family. The activation of the IRE1α/XBP1s signaling axis induces the expression of genes encoding proteins of the ERAD pathway (EDEM, HRD1) and factors that modulate protein translocation into the ER and folding, including the protein BiP [53,71]. Importantly, the non-spliced Xbp1 mRNA encodes the protein XBP1u (XBP1 unspliced), which is an inactive form with no transcriptional activity because it lacks the transactivating domain, and is an extremely short-lived protein. Interestingly, however, XBP1u was also found to interact with XBP1s under ER stress conditions, functioning as a negative feedback regulator [72,73].

IRE1α’s endoribonuclease activity has also been shown to induce rapid and specific degradation of some RNAs by a mechanism called RIDD (Regulated Ire1-Dependent Decay) (Figure 1) [74,75]. Currently, only a limited number of direct targets have been identified and validated, including 4 microRNAs (miR-17, 96, 125b, 34a) [76] and some mRNAs notably PER1 [77], SPARC [78], BLOS1 [79], and DR5 (death receptor 5), but bioinformatic studies coupled with transcriptomic studies suggest a wider spectrum of action [80,81]. Several studies indicate that the RIDD mechanism contributes to ER stress-induced cell death, notably by degrading several miRNAs involved in the repression of caspase-2 mRNA expression [75,76]. However, other studies propose that RIDD activity, by targeting mRNAs specifically translated at the endoplasmic reticulum, reduces the influx of newly synthesized proteins, and thus participates in the adaptive survival process [82]. Moreover, the IRE1α-mediated targeting through RIDD of the mRNA coding for the death receptor 5 protein, a cell surface transducer of apoptotic signals could also limit ER stress-induced cell death [83].

The IRE1α activation level, stability, conformation, and oligomerization status appear to be also regulated by the interaction with many different protein partners such as for example HSP47 which facilitates the dissociation of BiP from its luminal domain thus helping in activation of IRE1α signaling under low stress conditions [10,84,85].

IRE1α associates also with additional partners through its cytosolic domain to induce different signaling pathways. TRAF2 (TNF receptor-associated factor), an adaptor protein, associates with IRE1α’s kinase domain. The IRE1α/TRAF2 complex was found to interact with ASK1 (apoptosis signal-regulating kinase 1) to activate the c-Jun N-terminal kinase (JNK) and induce apoptosis [62,86]. Thus, the JNK arm of IRE1α pathway was initially thought to promote cell death. However, the function of this pathway in vivo is still controversial and has been described in some cases as pro-death and in other cases as pro-survival [62,86]. The nature and the intensity of the stimulus may account for these results. As in the case of ATF6α, IRE1α behaves as a sensor of the general cellular state through its multiple interactions with cofactors, regulators, and other members of the UPR signaling cascades and centralizes a set of signals in order to balance between anti- and proapoptotic signals.

Recent work has demonstrated that the activity of PERK and ATF6α can also be regulated by specific interacting proteins (reviewed in [10]). These results indicate that the activity of the three UPR effectors is extremely finely tuned. In addition, these effectors can establish crosstalk between each other during the UPR response and therefore more detailed analyses of these proteins and their identified partners remain necessary to better understand how they contribute on their own and altogether to the overall cell’s response during UPR activation.

## 3. Hematopoiesis and Leukemias

### 3.1. Hematopoiesis

**Figure 2:**
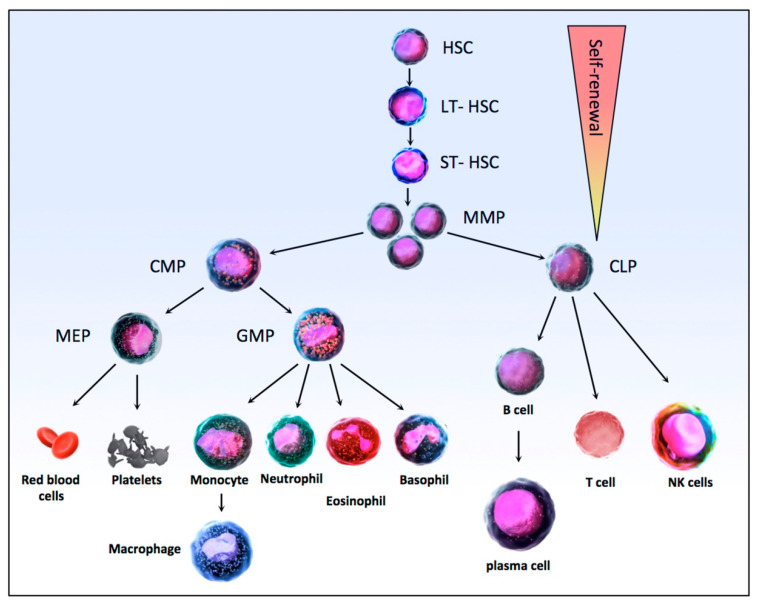
Schematic of the HSC differentiation hierarchy in normal hematopoiesis. HSC, Hematopoietic stem cells; LT-HSCs (Long-Term Hematopoietic Stem Cell) are able to generate new LT-HSC or to differentiate into ST-HSC (Short-Term Hematopoietic Stem Cell) then into MPPs (MultiPotent Progenitors) with reduced self-renewal capacity. Downstream of MPPs, a strict separation takes place between the myeloid (CMP, Common Myeloid Progenitors) and lymphoid (CLP, Common Lymphoid Progenitors) lineages. CMP then produce MEPs (Megakaryocyte–Erythrocyte Progenitors), which differentiate into platelets and erythrocytes, and GMPs (Granulocyte-Macrophage Progenitors) produce granulocytes (neutrophils, eosinophils, and basophils) and macrophages. In the lymphoid lineage, the CLPs then produce T and B lymphocytes and natural killer cells. The whole hematopoietic differentiation process is tightly regulated by a number of intrinsic and extrinsic factors, like cytokines and transcription factors.

Hematopoiesis is the physiological process that is responsible for the production of the mature pools of blood cells from undifferentiated precursors, the stem cells. Hematopoiesis, which takes place mainly in the bone marrow of long and flat bones, is a crucial process as it allows the maintenance of blood cell homeostasis, producing approximately 10^12^ blood cells daily in a healthy adult. The hematopoietic system functions as a pyramid-like hierarchy organized from a hematopoietic stem cell (HSC) at the top, able to self-renew or differentiate to produce all the cells of the hematopoietic system (Figure 2). In the bone marrow, long-term hematopoietic stem cells (LT-HSC) are quiescent, in the G0 phase of the cell cycle with a very low mitochondrial activity, but a high self-renewal potential [87]. These cells are maintained throughout life. In classical hematopoiesis, LT-HSC division leads to the generation of new LT-HSC or ST-HSC, for Short-Term Hematopoietic Stem Cell, which are able to produce all mature hematopoietic lineages [88,89]. These cells then differentiate into multipotent progenitors (MPPs) and then either in common lymphoid progenitors (CLPs) or in common myeloid progenitors (CMPs) [90]. CLPs produce B and T lymphocytes and natural killer cells, while CMPs generate granulocyte-macrophage progenitors (GMPs), which, as their name implies, are then differentiated into granulocytes and macrophages, and megakaryocyte–erythrocyte progenitors (MEPs) which themselves differentiate into red blood cells and platelets [91,92] (Figure 2).

Hematopoiesis is a tightly regulated mechanism, and therefore impaired hematopoiesis can be the cause of leukemias, malignant disorders resulting from defects of the stem cells at different stages of maturation, with subsequent clonal expansion [92]. Leukemias include acute and chronic leukemia and are also classified into lymphoblastic and myeloblastic leukemias according to the cell type affected. Acute leukemias are characterized by the proliferation of immature, unfunctional white blood cells called “blasts”, decreasing normal hematopoietic cells in the bone marrow while chronic leukemias are characterized by the expansion of differentiated cells in the blood [93]. Acute leukemias are divided into acute myeloid leukemias (AML) or acute lymphoblastic leukemias (ALL) and chronic leukemia into chronic myeloid leukemias (CML) or chronic lymphoblastic leukemias (CLL).

### 3.2. Acute Myeloid Leukemia (AML)

Acute myeloid leukemia (AML) is a group of phenotypically and genetically heterogeneous diseases, which is among the most common adult leukemia (it accounts for about 80% of leukemias in adults), with an average age of first diagnosis over 60 years [94]. This is a complex pathology triggered by the accumulation of chromosomal translocations and/or multiple mutations and resulting in the transformation and clonal expansion of hematopoietic progenitors. AML is thought to initially develop from at least two types of somatically acquired genetic alterations: mutations that confer advantages in terms of proliferation and survival and mutations that interfere with cell differentiation and apoptosis mechanisms [95]. Recent advances in sequencing methodologies have shown that AML represents a dynamic disorder in which multiple sub-clones compete and coexist, not only during the normal progression of the disease but also under pressure generated by anticancer agents [96]. While the majority of patients are in complete remission after the initial chemotherapy, AML has been associated with a poor prognosis because most patients tend to relapse due to the emergence of therapy-resistant clones [97]. Identifying the genetic alterations associated with resistance to chemotherapy is essential for risk stratification and to predict response to treatment of each AML patient. Three main classes of genetic aberrations have been described in AML: non-random chromosomal alterations, multiple gene mutations, and epigenetic alterations [98,99]. The most common of these chromosomal alterations include rearrangements leading to the formation of genes coding for chimeric proteins and upregulation of gene expression by juxtaposition with strong promoters. Among these rearrangements, we find translocations t(8;21) AML1-ETO or RUNX1, t(15;17) PML-RARA, inv(16) CBFB-MYH11, and t(9;11) MLL-AF9, which are associated with a better prognosis, whereas translocations t(11;19) MLL-ENL, t(6;11) MLL-AF6, t(10;11) MLL-AF10, or complex karyotypes are associated with a worse prognosis [100]. One of these translocations, t(15;17) (q22;q12), is peculiar because it is characteristic of a subtype of acute myeloid leukemia named acute promyelocytic leukemia (APL) [101]. This specific chromosomal translocation leads to the expression of the PML-RARα fusion protein. APL is unique among all leukemias because of its high level of sensitivity to all-trans retinoic acid (ATRA), the vitamin A acid form [102]. The prognosis of this pathology is very good in general [103,104]. Among the genes that have been found mutated in AML we can mention retinoic acid receptor-α (RAR-α), core binding factor (CBF), HOX gene family or MLL. Mutations in oncogenes such as FLT3, KIT, N-RAS, GATA-1, JUN B, MYC, p53, PU.1, RB, FES, FOS, MPL, WT1, WNT, CEBPA, and NPM1 or mutations affecting epigenetic modifiers such as DNMT3A, ASXL1, TET2, IDH1, and IDH2 have also been characterized [105,106,107].

### 3.3. Acute Lymphoblastic Leukemia (ALL)

Acute lymphoblastic leukemia (ALL), also called acute lymphocytic leukemia, is a rare genetically heterogeneous clonal malignant disorder of the bone marrow characterized by immature lymphoid precursors proliferation leading to the crowd out of normal hematopoietic cells [108]. ALL, which can occur at virtually any age, is more frequently seen in children and adolescents. This pathology results from clonal proliferation of abnormal B cell progenitors (B-ALL) accounting for approximately 85% of ALL or T cell progenitors (T-ALL) accounting for roughly 15% of ALL. Most of the genetic alterations leading to leukemogenesis, including chromosomal translocations, somatic mutations, aneuploidy, and gene copy number alterations have been characterized in both T-ALL and B-ALL. Like in AML, these genetic alterations are important prognostic factors for disease-risk stratification and treatment [109,110]. Among the genetic alterations found in B-ALL, TCF3–PBX1 t(1;19), ETV6–RUNX1 t(12;21), and hyperdiploidy are associated with a favorable outcome while MLL rearrangements, TCF3–HLF t(1;19) and rearrangements of CRLF2, JAK2A, or BL-class tyrosine kinase genes are of poor prognosis [111,112]. Alterations involving the KRAS, NRAS, FTL3, PTPN11, and epigenetic modifiers like CREBBP or WHSC1 are frequent genetic events [111,113].

The genetics of T-ALL is extremely heterogeneous, with chromosomal abnormalities in nearly all patients. Mutations in the NOTCH1 gene leading to constitutive activation of NOTCH signaling is the main oncogenic pathway found in the majority of patients. These alterations are generally associated with loss of p16 (INK4A) and p14 (ARF) suppressor genes at the CDKN2A locus. In addition, in 50% of patients with T-ALL, chromosomal translocations affect genes encoding oncogenic transcription factors like TAL1, TAL2, MYC, MYB, LYL1, TLX1 (HOX11), TLX3 (HOX11L2), or HOXA genes, placing these genes under the control of powerful T cell specific activators [114].

As for the AML example, it is not possible to exhaustively list all the genetic alterations and different combinations encountered, so we refer the reader to references dealing more specifically with this pathology [115,116,117].

### 3.4. Chronic Myeloid Leukemia (CML)

Chronic myeloid leukemia (CML) is a slow-growing myeloproliferative neoplasm characterized in more than 95% of cases by the t(9;22) (q34.1;q11.2) chromosomal translocation leading to the formation of the Philadelphia chromosome (Ph*), resulting in the BCR-ABL1 gene fusion. The subsequent BCR/ABL1 chimeric protein is a constitutively active tyrosine kinases oncoprotein which activates transduction pathways involved in cell growth and differentiation such as RAS, MYC, STAT, AKT RAF, or JUN, and is therefore capable of transforming hematopoietic stem cell into neoplastic one [118,119].

Before targeted therapies became available, the main treatment options for CML included allogeneic stem cell transplantation and chemotherapy. However, the prognosis for CML improved considerably since the use of tyrosine kinase inhibitors (TKIs), most notably imatinib in the early 2000s, which inhibit the BCR-ABL1 fusion protein by blocking its kinase domain [120]. Several generations of TKI have been developed since, but the appearance of TKI resistances remains a major issue [121]. It is therefore also crucial for this pathology to identify new therapeutic approaches in order to better stop its progression and avoid evolution to advanced disease states which may account for as much as 15% of all CML deaths [121].

### 3.5. Chronic Lymphocytic Leukemia (CLL)

Chronic lymphocytic leukemia (CLL) is characterized by a clonal proliferation and accumulation of mature but defective lymphocytes in the blood, bone marrow, lymph nodes, and spleen. CLL is the most common form of leukemia in Western countries. It is highly heterogeneous in its evolution, with some patients needing chemotherapy early after diagnosis and others never requiring specific treatment and having a survival rate similar to the general population. More than 95% of people with CLL develop the B cell type [122]. CLL is a heterogeneous disease, which divides into an aggressive form that expresses a wild type immunoglobulin heavy-chain variable region (IGVH) gene, and an indolent form that expresses a mutated IGVH, reflecting the stage of normal B cell differentiation [123,124]. Chronic lymphocytic leukemia cells exhibit many complex genetic alterations, which have been used by clinicians as prognostic biomarkers in order to predict survival and disease progression and guide treatment decisions [124]. Many recurrent cytogenetic abnormalities are encountered in CLL. The main ones are (i) deletion of the long arm of chromosome 13 (del(13q)), leading to the loss of the DLEU2/MIR15A/MIR16-1 genes, which is found in more than 50% of CLL cases and is of good prognosis when isolated; (ii) trisomy 12, associated with an intermediate prognosis with median overall survival; (iii) deletion of the long arm of chromosome 11 (del(11q)) that leads a more aggressive disease due to the loss of the ATM gene (for Ataxia Telangiectasia Mutated) which is essential for the regulation of the cell cycle; and (iv) deletion of the short arm of chromosome 17 (del(17p)) resulting in the loss of the TP53 gene which is of poor prognosis [125,126,127]. At least one of these abnormalities can be found in approximately 80% of patients [122,128,129]. Translocations are reported in approximately 20% of CLL [130]. These translocations predominantly involve the immunoglobulin genes, mainly IGH, and the 13q14 locus. Common partners are CCND1, BCL2, and BCL3 [130]. In addition to chromosomal rearrangements, sequencing studies have also revealed numerous recurrent mutations in CLL mostly in the P53, ATM, NOTCH1, SF3B1 (Splicing Factor 3B subunit 1), and BIRC3 genes [131].

A variety of targeted drugs including BCR signaling pathway inhibitors, anti-CD20 antibodies and BCL-2 inhibitors have been used in therapeutics and have significantly improved the management of this disease [132,133]. However, despite the increasing number of available therapeutic alternatives, chemotherapy does not currently provide a definitive cure and additional strategies are still required.

## 4. Endoplasmic Reticulum Stress Induction in Hematopoietic and Leukemic Cells

### 4.1. ER Stress Activation in HSCs

Hematopoietic stem cells (HSCs) are sitting at the apex of the hematopoietic hierarchy. They are the most immature cells and are capable of replenishing all hematopoietic cell types [134,135]. As long-life cells, HSCs require a highly regulated protein quality control in order to avoid the accumulation of damages that could ultimately affect their DNA integrity and promote tumorigenesis. At steady state, HSCs are quiescent and display lower protein synthesis rates in vivo and in vitro compared to their progeny [136]. Furthermore, HSCs have been associated with low protein folding capacity that can be explained by a lower expression of chaperones proteins compared to hematopoietic progenitors [137]. Moreover, protein synthesis deregulation has a great impact on HSCs’ viability and self-renewal capacities and can lead to HSCs loss [136,138]. Investigation of ER stress role in regulating hematopoietic stem cells fate, revealed a high expression of PERK and a low expression of eIF2α in HSCs when compared to progenitor cells [139,140]. PERK upregulation in HSCs appears to increase their sensitivity to ER stress, compared to more committed progenitor cells, through activation of the PERK-peIF2α-ATF4/CHOP arm that can trigger apoptosis. It has been suggested that this sensitivity to ER stress could prevent accumulation of damaged cells in the HSCs compartment and potential subsequent malignant transformation [139]. In agreement with this hypothesis, Miharada et al. showed that reducing ER stress levels in vitro in HSCs through the overexpression of the RNA binding protein Dppa5 (Developmental pluripotency-associated 5) improved their self-renewal activity by protecting them from apoptosis [141]. However, the IRE1α-XBP1 UPR branch can also be activated in HSCs and in this case plays a significant cytoprotective role. For example, estrogen treatment of HSCs activates the IRE1α-XBP1 branch and increases repopulation capacities of HSCs upon transplantation [142]. In a mouse model system, Liu et al. also showed that IRE1α-XBP1 activation in HSCs in vivo prevents ER stress-induced apoptosis, preserves HSC clonogenicity and improves reconstitution capacity [143]. Xie et al. also demonstrated that increased cytoprotective ER stress (induced by the pharmacological inhibition of the sphingolipid enzyme DEGS1) participates together with autophagy in the setting up of a prosurvival response aimed to maintain stemness properties [144].

Increased ERAD has also been recently reported to actively participate in the maintenance of proteins homeostasis in HSCs and appeared to be essential for stem cell pool maintenance [145]. In addition to low protein synthesis rates and low folding capacity, it has been reported that protein quality control by ERAD maintains HSCs pool. Altogether currently known data indicate that increased basal UPR induced at least in part by unfavorable growing conditions in the bone marrow environment, such as, e.g., hypoxia [146], helps in maintaining HSC integrity as well as clearing damaged HSCs and therefore play critical functions at the early steps of hematopoiesis [147]. Of note, in our article we refer to “basal UPR” as the activation status of the different signaling pathways of the UPR in cells growing either in vitro or in vivo without any treatment by chemotherapeutic drugs or chemical compounds.

### 4.2. ER Stress Activation in Leukemic Cells

Recent lines of evidence link activation of the three UPR branches to most hallmarks of cancer and especially those aimed to protect the cells against the numerous aggressions they undergo during their growth inside tumors [20]. This is especially true for solid tumors, which develop in a highly adverse environment but also for leukemic cells. Indeed, hematopoietic cells, either normal or leukemic, are exposed in the bone marrow to an adverse environment caused by hypoxia, high levels of reactive oxygen species (ROS), and nutrient deprivation, often resulting in ER stress activation [13,16,23,148]. Thus, many studies have reported the activation, to variable extents, of each of the three UPR branches (IRE1α, PERK, and ATF6α) in a wide range of hematopoietic tumors (leukemia, lymphoma, and myeloma) [137,149,150,151]. As in solid cancers, UPR plays a fundamental role in the adaptation of leukemic cells to cellular stress by inducing different mechanisms, which attempt to reestablish ER homeostasis in order to restore its proper functions.

In AML patients, increased expression of XBP1, BiP, and Calreticulin has been detected in 17.4% of cases [152]. Schardt et al. demonstrated a correlation between a high expression of XBP1s and complex karyotype in AML [152]. Another clinical study from Tanimura et al. reported activation of the IRE1α-XBP1 pathway in AML patients; however, no significant correlation between ER stress activation and genetic features could be revealed [153].

Interestingly, UPR activation in some hematological malignancies is not always the consequence of stress integration but can also be induced through aberrant pathway activation. For example, in chronic lymphoid leukemia (CLL), UPR activation is observed in response to surface immunoglobulin M stimulation and activation of the kinases BTK and SYK [154]. In pre-B-ALL, Xbp1 expression is activated by various oncogenic tyrosine kinases via STAT5 signaling [155]. Moreover, the transcription factor c-Jun, overexpressed in AML and CML, promotes the transcription of general UPR target genes such as Xbp1 and Atf4 by a direct mechanism [156]. The modulation of expression of some UPR effectors in leukemia has been shown to involve epigenetic modifications in their promoters [157,158].

**Figure 3:**
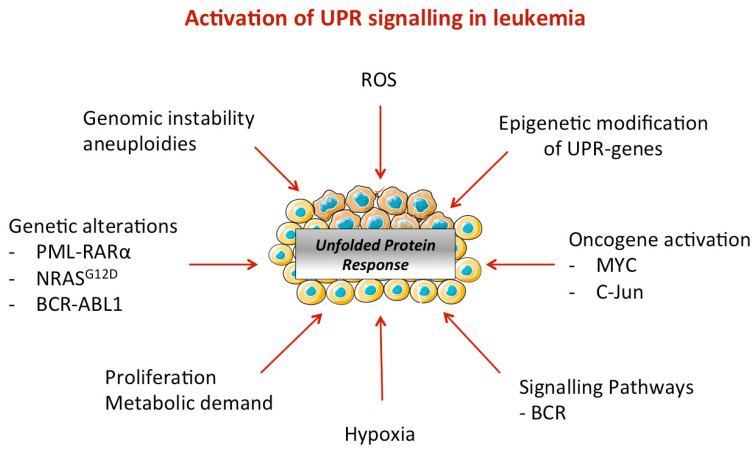
Activation of UPR signaling in leukemia. Different mechanisms of ER stress activation have been reported in leukemia, which include (epi)genetic modifications and genomic instability (e.g., mutations, translocations, hypomethylation), oncogenic signaling, and metabolism rewiring due to a high proliferation in blasts. Microenvironment is also a well-known source of ER stress (e.g., hypoxia) that contributes to UPR activation.

In addition, mutations in epigenetic splicing factors, which are considered as first hit mutations, have pleiotropic effects that might be linked to ER stress activation (Figure 3). The comparison between healthy donor and AML patient samples revealed hypomethylation of Xbp1′s promoter that has been suggested to lead to overexpression of XBP1. On the contrary, in large diffuse B cells lymphoma, IRE1α expression is reduced through a mechanism involving the histone methyltransferase, EZH2 (Enhancer of Zeste Homolog 2).

Furthermore, transcription of ER stress-related proteins by oncogenic pathways also participates in the UPR activation in leukemia. For instance, the MAPK pathway promotes the transcription of Xbp1 through STAT5 activation. In pre-B acute lymphoblastic leukemia (ALL), BCR-ABL1 or NRASG12D signals through MAPK-STAT5-XBP1 [155]. Indirectly, in lymphomagenesis the transcription factor MYC, by promoting a rapid cell proliferation, increases the rate of misfolded proteins in ER that triggers the UPR [159].

Compared to HSC, in which both cytoprotective (IRE1α-XBP1) and cell death-promoting (PERK) UPR pathways can be activated in the basal state, the activation of an adaptive UPR (mainly via IRE1α signaling) appears to be preferred in leukemic cells. However, the increase in basal UPR could sensitize these cells to additional stress induced, for example, by chemotherapy treatment (see next chapter). By analogy with what is observed in HSCs under normal growth conditions, the UPR response may represent a real checkpoint influencing cell fate of leukemic cells experiencing chemotherapy: either the stress can be resolved via an adaptive phase and cancer progresses or the damage accumulates and becomes unrecoverable. In this latter case, excessive or prolonged stress triggers proapoptotic signaling through a terminal process [137,150,151,160]. This important issue is discussed below.

### 4.3. UPR Modulation: A Double-Edged Sword to Fight Against Leukemia

Despite the numerous pieces of evidence of reticulum stress activation in multiple cancers, the question of whether UPR reduces or promotes tumor growth in patients is still the subject of intense debate [149]. Two therapeutic strategies exploiting ER stress and UPR could be possible in order to induce leukemic cell death: either inhibition of the adaptive UPR response (cytoprotective) or activation of the terminal UPR response (cytotoxic). The choice between these two strategies may be difficult as their relative efficacy may be highly dependent on the cellular deregulation that led to the disease.

As mentioned above, various studies have shown that leukemic cells often possess basal UPR activity with a cytoprotective function, which favors tumor progression and additionally may increase chemoresistance of the cells to various drugs. For example, the ER stress sensor BiP was found to be highly expressed in B-ALL and its pharmacological inhibition by epigallocatechin gallate (a polyphenolic compound purified from green tea) sensitized cells to the anti-leukemic drug vincristine [161]. In the same pathology, the increase in expression and activity of BiP and the IRE1α/XBP1 pathway were found to be essential for cell survival and pharmacological inhibition of IRE1α RNAse domain by the drug STF-083010 reduced the proliferation and survival of patient-derived pre-B ALL cells [155]. Of note, increased Xbp1 mRNA levels at diagnosis appear of poor prognosis for patients with the disease [155]. In CML, activation of the PERK-eIF2α pathway has a cytoprotective effect and increases their resistance to imatinib, a tyrosine kinase inhibitor widely used in cancer chemotherapy [162]. Resistance to imatinib in CML was also shown to result from the activation of ATF6α, which appears mediated by the protein disulfide isomerase 5 (PDIA5) upon ER stress and a PDIA5 inhibitor, 16F16, increased cells’ sensitivity to treatment with imatinib [163]. Moreover, a pharmacological inhibitor of IRE1α, B109, was reported to suppress CLL tumor cell progression in a murine model and to sensitize human CLL cells to the Bruton’s tyrosine kinase (BTK) inhibitor ibrutinib [164]. A pro-survival role for IRE1α was also reported in AML and the pharmacological inhibition of IRE1α by 2-hydroxy-1-naphthaldehyde (HNA) switched the cells towards apoptosis and in addition synergized with treatments with bortezomib and arsenic trioxide, two widely used anticancer drugs [157]. Moreover, analysis of Philadelphia chromosome (Ph)-positive AML patient samples revealed increased expression of the BiP, CHOP, and Xbp1s mRNAs and the authors demonstrated that inhibition of the IRE1α and ATF6α pathways sensitized cells expressing the Bcr-Abl fusion protein to imatinib- and etoposide-induced apoptosis [165]. More recently it was shown that Jun itself induces the expression of several UPR effectors thereby enhancing UPR induction and this appeared essential to AML cell proliferation and survival, thus demonstrating that Jun could contribute to induce an adaptive UPR in some AML subtypes [156].

Altogether, the data presented above have largely validated the inhibition of adaptive UPR as an effective means of fighting leukemia and a significant number of pharmacological inhibitors of central UPR effectors are currently under preclinical studies or clinical trials [137,150].

However, these promising results should not lead us to neglect the other strategy aimed at inducing a cytotoxic response in the cell through terminal UPR induction, the “second edge of the sword”. Indeed, leukemic cells, which usually experience unfavorable growth conditions and maintain increased levels of ER stress and basal UPR, may show an increased susceptibility to enter terminal UPR in response to different treatments. Indeed, artificially increasing the unfolded protein load can lead to a cytotoxic cellular response in some leukemic models. Thus, in ALL treatment with the drug pevonedistat, which inhibits the NEDD8 conjugation pathway and impairs degradation of misfolded proteins by the proteasome, induces a reorientation of UPR towards apoptosis [166]. Interestingly, inhibiting the ER-associated degradation (ERAD) pathway of proteins by knockdown of one of its components, UFD1, also results in the induction of a terminal UPR process in T-ALL cells in response to the accumulation of unfolded proteins [167]. In mast cell leukemia, it was demonstrated that moderate pharmacological inhibition of IRE1α could stop leukemic cell proliferation by impairing adaptive UPR but with non-significantly induced cell death. Interestingly, stronger inhibition of IRE1α induced a switch from adaptive to terminal UPR. Enhancing ER stress by pharmacological inhibition of proteasome activity with bortezomib also induced terminal UPR in this model [168]. In Philadelphia-positive ALL, pharmacological inhibition of IRE1α with MKC-8866 also appeared able to reorient the initial cytoprotective UPR program towards cell death induction when combined with the inhibition of BCR-ABL1 with nilotinib [169].

Few studies describing the induction of cell death in leukemic cells by a strategy deliberately aimed at redirecting the cell response towards terminal UPR has yet been described. However, the analysis of data published over the last two decades and describing the use of antileukemic drugs shows, strikingly, that for many of them (see Table 1) their mode of action involves the induction of a terminal UPR pathway or related UPR-induced cell death processes. Indeed, although adaptive UPR was found to contribute to chemoresistance in 10 out of the 91 chemical compounds tested against leukemic cells and listed in Table 1, for the remaining compounds (i.e., 89% of the whole) the induction of UPR signaling pathways was associated with cytotoxicity. There appears to be no apparent correlation between the type of leukemia and the final response, cytoprotective or cytotoxic, to UPR induction. This also seems to be the case if we consider the mode of action of the drugs used. Similarly, no strict correlation can be found between the UPR pathways activated in response to the drugs and the final response of the cell (pro-survival or pro-death) and all UPR pathway have been reported to be induced whatever the final outcome on leukemic cell’s viability. It can be noted, however, that the CHOP pathway is very frequently activated when UPR induction results in cell death. This appears not surprising as the PERK-peIF2α/ATF4/CHOP signaling pathway plays a crucial function in inducing cell apoptosis in the cell [46] and was reported to be a major cell death-inducing UPR pathway in hematopoietic stem cells, as described above (see Chapter 4.1). However, induction of the PERK-peIF2α/ATF4/CHOP signaling pathway can also be detected in leukemic cells responding to treatment by an adaptive UPR. As in other pathological models, leukemic cell response to UPR induction is a complex process, which may rely on a subtle balance between the activation levels of the different branches of the UPR. Anyway, it appears that for a large number of chemotherapeutic agents or candidate compound, this process is critical for the final death/survival outcome of leukemic cells.

Therefore, it seems important to further investigate terminal UPR induction, on its own as well as in combination with other pharmacological treatments, for the improvement of therapeutic strategies in leukemia.

## 5. Conclusions

We have reviewed the currently available data in the literature dealing with the various roles played by UPR in leukemia. We also presented some of the UPR-mediated molecular processes that can induce cytoprotection of leukemic cells or direct them towards cell death through apoptosis induction. In the light of all of the currently reported data from studies carried out to dissect the role of UPR in the progression of leukemia, it is clear that the cytoprotective/cytotoxic balance regulation is a complex, highly dynamic machinery, still poorly understood and that a wide and integrative approach is needed to discover the genuine mechanisms underlying this crucial process. The specific networks that regulate ER stress-induced cytoprotection or apoptosis may be dependent on the nature, the intensity and the length of the stimuli. It is probable, even if contradictory results have sometimes been published, that it also depends on the cell type being stressed.

A better understanding of the UPR mechanisms acting in response to chemotherapy appears also essential to provide new therapeutic pathways aimed to eradicate neoplastic cells either by inhibiting the adaptive UPR, or by activating UPR-mediated cell death pathways [265].

## Figures and Tables

**Table 1:** Consequences of UPR induction by drugs in leukemic cells. For each drug, the chemical nature, the pubchem compound ID, and the molecular target are provided when available. The type of leukemia on which the work was carried out was provided as well as the inferred or demonstrated role of UPR activation. The involved effectors are also indicated, when they have been identified (n.d.: not determined).

Molecule	Chemical Nature	Pubchem Compound CID	Target	Type of Leukemia	Proposed Role of ER Stress/UPR Activation	Mainly Implicated Pathways/Effectors	Ref.
BIX-01294		25150857	Histone methyltransferase G9A	AML	Adaptive	PERK (prosurvival, via NRF2)	[170]
SCH727965 (Dinaciclib)		46926350	CDKs 1,2,5 and 9	AML, CML, T-ALL	Adaptive	XBP1s	[171]
Ski, ROMe		16760659	spingosine kinases 1 and 2	T-ALL	Adaptive	unclear	[172]
MDA-7/IL-24)	cytokine	-	n.d.	AML, APL	Adaptive	GRP78/Bip, IRE1α, GADD34	[173]
sorafenib	multikinase inhibitor	216239	MEK/ERK pathway	U937 cell line	Adaptive	PERK	[174]
Digoxine	cardiac glycoside	2724385	Na+/K+ ATPase	K562 (erythroleukemic) and THP-1 (acute monocytic leukemia) cell lines	Adaptive	PERK, IRE1α	[175]
Shikonin	naphthoquinone	479503	pyruvate kinase-M2 (PKM2), proteasome inhibition, NFkB, Thioredoxin reductase (TrxR1)	HL60	Adaptive	ERP57 and Calreticulin	[176]
3-deazaneplanocin A (DZNeP)	cyclopentenyl analog of 3-deazaadenosine	73087	histone methyltransferase	MV4-11, MOLM-14, Mono-Mac-1, THP-1, HL60 and KG-1 AML cell lines	Adaptive	GRP78, GRP94, PERK, PDIA isoform 3, 4, and 5	[177]
wolfberry phytochemicals	n.d.	-	n.d.	Jurkat cell line	Adaptive	all UPR pathways	[178]
Imatinib		5291	tyrosine kinases inhibitor	CML/LAMA-84 CML cell line and murine myeloid progenitor primary cells	The constitutive activation of PERK in CML cells protects from imatinib treatment	PERK	[162]
Metformin		4091	multiple, see PMID: 28776086	T-ALL, B-ALL	Switch form adaptive to terminal	IRE1α, CHOP	[179]
Nilotinib + MKC8866		644241	Tyrosine kinases (Nilotinib); IRE1α (MKC8866)	ALL (Ph+)	Switch form adaptive to terminal	IRE1α (cytoprotective); PERK and ATF6α (cytotoxic)	[169]
2-deoxy-D-glucose	glucose analog	108223	n.d.	ALL Cell Lines	Switch form adaptive to terminal	GRP78/Bip, CHOP	[180,181]
Selenite	sodium selenite	24934	n.d.	NB4 cell line (APL)	Switch form adaptive to terminal	PERK/eIF2α/ATF4	[182]
Asperuloside	iridoid glycoside	84298	n.d.	cell lines HL60 and U937, primary leukemic cells	Terminal: apoptosis induction	all UPR pathways	[183]
JA3 & JA7	Aldehyde biphenyl chalcones	134820953	n.d.	(AML); T-ALL; CML	Terminal: immunogenic apoptosis-like cell death	CHOP; PERK	[184]
Oprozomib	tripeptide analog of carfilzomib	25067547	immunoproteasome subunit β5i/LMP7 (ubiquitin–proteasome pathway)	CML	Terminal: apoptosis induction	PERK, IRE1α (via ASK/JNK/Bim)	[185]
VAS3947	given in [186] fig 1a	7471335	NADPH oxidases	AML	Terminal: apoptosis induction	IRE1α, PERK	[186]
Arsenic trioxide + Gilteritinib		14888/49803313	FLT3 (for Gilteritinib)	AML (FLT3-ITD)	Terminal: apoptosis induction	IRE1α	[187]
GSK-J4		71729975	H3K27me3 demethylase	AML	Terminal: apoptosis induction	PKCα; Bcl2 phosphorylation	[188]
CXL146	4H-chromene derivative	-		AML (or APL): HL60; CML	Terminal: apoptosis induction	PERK, IRE1α, ATF6α	[189]
FF-10501	given in paper	124343	inosine monophosphate deshydrogenase	AML	Terminal: necrotic cell death	CHOP	[190]
Retinoic acid+Tunicamycin+ arsenic trioxide		444795/11104835	n.d.	AML	Terminal: Cytotoxic UPR	CHOP, XBP1s	[191]
[Retinoic acid or arsenic trioxide] + tunicamycin		444795/11104835	n.d.	APL	Terminal: Cytotoxic UPR		[192]
MIM1 and UMI-77		135691163/992586	n.d.	AML; T-ALL	Terminal: Cytotoxic UPR	NOXA	[193]
PFR	peptide	-	n.d.	AML	Terminal: necroptosis		[193]
Genistein	Isoflavone	5280961	n.d.	AML or APL (HL60 cell line)	Terminal: Cytotoxic UPR		[194]
Camalexin	Phytoalexin (structure given in paper)	636970	n.d.	AML	Terminal: apoptosis induction	PERK, CHOP	[195]
Ibrutinib (PCI-32765)		24821094	Bruton’s tyrosine kinase	B-ALL	Terminal: apoptosis induction	ATF4; CHOP	[196]
OT-55	bis-coumarine derivative	-	n.d.	CML	Terminal: Immunogenic cell death induction; apoptosis induction	not well documented	[197]
RS-F3	fistularin-3 stereoisomer	-		AML	Terminal: Cytotoxic UPR (assumed)	PERK; XBP1s; CHOP	[198]
Bardoxolone methyl (CDDO-Me)	triterpenoid	400769	Nrf2 and NF-κB	Chronic myeloid leukemia, K562 cell line	Terminal: apoptosis induction	PERK, IRE1α, CHOP	[199]
3-O-trans-p-coumaroyl-alphitolic acid (3OTPCA)	triterpenoid	-	n.d.	U937, Molt-4 and Jurkat cell lines.	Terminal: apoptosis induction	XBP-1 and CHOP	[200]
Nelfinavir		64143	HIV protease inhibitors	T-ALL, B-ALL, and AML; CLL primary leukemic cells	Terminal: apoptosis induction; In CLL, contributes to the induction of cell death in choroquine treated cells	CHOP	[201,202]
CB-5083	1-[4-(benzylamino)-5H,7H,8H-pyrano[4,3-d]pyrimidin-2-yl]-2-methyl-1H-indole-4-carboxamide	439268	Valosin-Containing Protein/p97	B-ALL Cell Lines (BALL1, REH, NALM6, OP1, ALL-PO, 697, RS4;11, BV173, SEM, and SUPB15)	Terminal: apoptosis induction	all UPR pathways	[203]
Tunicamycin ± Quizartinib (AC220)	AC220	11104835 + 24889392	FLT3	AML (FLT3-ITD)	Terminal: apoptosis induction	PERK, CHOP	[204]
Cryptotanshinone	lipophilic diterpene quinone	160254	n.d.	CCRF-CEM cell line (ALL)	Terminal: apoptosis induction	IRE1α-XBP1, PERK-eIF2α-ATF4	[205]
Oxalicumone A	dihydrothiophene-condensed sulfur chromone	90676613	n.d.	KG-1a, HL60, U937, and K562 cell lines (AML)	Terminal: apoptosis induction	IRE1α-XBP1, PERK--CHOP	[206]
Pevonedistat (MLN4924)	adenosine sulfamate analog	16720766	NEDD8-activating enzyme	T-ALL (CCRF-CEM, Jurkat) and B-ALL (REH, NALM6, SupB15) cell lines	Terminal: apoptosis induction	all UPR pathways	[166]
Carfilzomib (PR-171)	tetrapeptide epoxyketone	11556711	ubiquitin–proteasome pathway	CLL MEC1 and MEC2 cell lines and primary leukemic cells	Terminal: apoptosis induction	ATF4, CHOP	[207]
Miltirone	abietane-type norditerpenoid quinone	160142	n.d.	Jurkat, U937, AML and ALL primary leukemic cells	Terminal: apoptosis induction	PERK	[208]
Arsenic trioxide		14888	n.d.	NB4 cell line (AML)/CML	Terminal: apoptosis induction	IRE1α-XBP1/GRP78/Bip, CHOP, Xbp1 (unspliced…)	[209,210]
JPH203	O-[(5-Amino-2-phenyl-7-benzoxazolyl)methyl]-3,5-dichloro-L-tyrosine dihydrochloride	24853505	LAT1 (L-type amino-acid transporter 1)	Ke37, DND41, Sil-ALL, Peer, Molt-16, Jurkat and SupT1 T-ALL cell lines	Terminal: apoptosis induction	CHOP	[211]
Wogonin	5,7-dihydroxy-8-methoxyflavone	5281703	n.d.	HL-60 cell line.	Terminal: apoptosis induction	all UPR pathways-CHOP	[212]
Farnesol	acyclic sesquiterpene alcohol	445070	n.d.	Molt4 T-ALL cell line	Terminal: apoptosis induction	PERK-eIF2α-ATF3/4	[213]
APO866	(E)-N-[4-(1-benzoylpiperidin-4-yl)butyl]-3-pyridin-3-ylprop-2-enamide	6914657	nicotinamide phosphoribosyltransferase (NAMPT)	OCI/AML2, OCI/AML3, HL-60, HEL, KG1a, SET1, MV4-11, MEC.1, MEC.2, LAMA-84 cell lines and B-CLL and AML primary leukemic cells	Terminal: apoptosis induction	IRE1α-CHOP	[214]
Bortezomib	boronic acid	387447	proteasome (26S)	NB4 cell line (APL)	Terminal: apoptosis induction	Nd	[215]
CX-4945	5-(3-chloroanilino)benzo[c][2,6]naphthyridine-8-carboxylic acid	24748573	casein kinase 2	T-ALL cell lines and primary cells	Terminal: apoptosis induction	GRP78/BIP-IRE1α-CHOP	[216]
compound 3 (Pyrimidine analogue)	1-(5,5,5-trichloro-2-methoxy-4-oxopenten-2-yl)-4-trichloromethyl-pyrimidin-2(1H)-one	-	n.d.	L1210, CEM, JURKAT cell line (ALL)	Terminal: apoptosis induction	CHOP and caspase-12	[217]
R7, R13	Naphtylchalcones		n.d.	murine lymphoblastic leukemia	Terminal: apoptosis induction (assumed)	CHOP	[218]
S1 (BH mimetic)				APL	Terminal: cytotoxic through NOXA induction	PERK; XBP1s, NOXA	[219]
Gossypol (BH3 mimetic)	polyphenol	3503	phospholipase A2	AML, APL	Terminal: cytotoxic through NOXA induction	PERK; NOXA	[220]
Cariporide		151172	Na + H+ exchanger 1 (NHE1)	CML, APL, T-ALL	Terminal: sensitizes to extrinsic, TRAIL-induced, apoptosis	CHOP	[221]
Auranofin		6333901	thioredoxin reductase	CLL	Terminal: contributes to the induction of cell death in treated cells	PERK; XBP1s, CHOP	[222]
[Cu(thp)4][PF6]	phosphine copper(I) complex		n.d.	B-ALL	Terminal: apoptosis induction	Xbp1s, CHOP	[223]
Z-Leu-Leu-Nle-CHO	leupeptin analog	-	γ-Secretase	CLL primary leukemic cells	Terminal: apoptosis induction	IRE1α, CHOP	[224]
curcumin	Diferuloylmethane	969516	n.d.	WEHI-3 myelomonocytic leukemia cell line/NB4 and UF-1 APL cell lines/HL60 cell line	Terminal: apoptosis induction	IRE1α, ATF6α, CHOP/PERK, CHOP, ASK	[225,226,227]
LQB 118	pterocarpanquinone	46233300	n.d.	K562 and Jurkat cell lines	Terminal: apoptosis induction (assumed)	caspase 12	[228]
Flavopiridol	Flavonoid alkaloid	5287969	CDKs inhibitor	CLL primary leukemic cells	Terminal: contributes to the induction of cell death in choroquine treated cells	IRE1α/XBP1 and CHOP	[229]
Safrole		5144		AML (HL-60)	Terminal: apoptosis induction (assumed)	ATF6α, CHOP	[230]
Clofibrate		2796	peroxisome proliferator-activated receptor (PPAR) alpha	T-ALL	Terminal: apoptosis induction (assumed)	ASAPK/JNK	[231]
Abnobaviscum F ^®^	Mitletoe aqueous extract	135343633	n.d.	CML	Terminal: contribution to the induction of cell death in treated cells (assumed)	GRP78/Bip, CHOP	[232]
MJ-29	Quinazolinone	-	n.d.	murine myelomonocytic leukemia	Terminal: apoptosis induction	GRP78/Bip, CHOP, PERK	[233]
Imatinib (STI571)		5291	BCR-ABL tyrosine kinase		Terminal: apoptosis induction	not well documented	[234]
glycyrrhizic acid		14982	n.d.	murine myelomonocytic leukemia	Terminal: contributes to the induction of cell death in treated cells	GRP78/Bip, CHOP	[235]
Gypenosides		-	n.d	HL-60 AML cell line	Terminal: apoptosis induction	ATF6α and ATF4	[236]
AICAr (+ methotrexate)	5-aminoimidazole-4-carboxamide (AICA) riboside	266934	n.d	Nalm6 and CCRF-CEM cell lines (ALL)	Terminal: apoptosis induction	CHOP (C/EPB homologous protein)	[237]
Emodin	6-methyl-1,3,8-trihydroxyanthraquinone	3220	n.d.	WEHI-3 murine myelomonocytic leukemia cell line	Terminal: apoptosis induction (assumed)	n.d.	[238]
Syrbactin	azamacrocyclic product	-	proteasome (26S)	REH ALL cell line	Terminal: apoptosis induction	CHOP (C/EPB homologous protein)	[239]
ABT-737 and GX15-070	BH3 mimetics	11228183/46930997	BCL2 family proteins	Jurkat, NB4 and K562 cell lines	Terminal: Cytotoxic UPR	ATF4, ATF3, CHOP and NOXA,	[240]
NPB001-05	n.d.	-	BCR-ABL	K562 cell line	Terminal: apoptosis induction (assumed)	not well documented	[241]
Ras inhibitor farnesylthiosalicylic acid (FTS, Salirasib)	2-[[(2E,6E)-3,7,11-trimethyl-2,6,10-dodecatrien-1-yl]thio]-benzamide	5469318	RAS	K562 cell line	Terminal: Cytotoxic UPR	not well documented	[242]
PYZD-4409	3,5-dioxopyrazolidine compound, 1-(3-chloro-4-fluorophenyl)-4-[(5-nitro-2-furyl)methylene]-3,5-pyrazolidinedione	60111983	ubiquitin-activating enzyme UBA1	K562, NB4, THP1, and U937 cell lines and AML primary leukemic cells	Terminal: apoptosis induction	PERK, CHOP, ATF4	[243]
Korbazol	n.d.	-	n.d.	CLL primary leukemic cells	Terminal: apoptosis induction (assumed)	n.d.	[244]
Polymethoxyflavone tangeretin (TAN)	Flavonoids	-	n.d.	K562 cell line	Terminal: apoptosis induction	IRE1α, PERK, CHOP	[245]
Shiga toxine type 1 (Stx1)	n.d.	-	ribosomes (protein synthesis)	THP-1 cell line	Terminal: apoptosis induction	CHOP, TNF-related apoptosis-inducing ligand (TRAIL), DR5 and calpain	[246]
Eicosapentaenoic acid		446284	n.d.	HL60 (AML or APL)	Terminal: apoptosis induction (assumed)	PERK	[247]
Xanthohumol	prenylated chalcone	639665	n.d.	CLL (patient samples)	Terminal: apoptosis induction	PERK, CHOP	[248]
Tunicamycin (UPR inducer)		11104835	N-acetylglucosamine phophotransferase	AML (U937 and HL60)	Terminal: cytotoxic through induction of lysosomal apoptotic pathway	GRP78/Bip, CHOP	[249]
arsenic sulfide	[As4S4 (AS)]	61569	n.d.	BCR/ABL-positive K562 cell line	Terminal: apoptosis induction	not well documented	[250]
Fenretinide	synthetic retinoid derivative (related to vitamin A)	5288209	n.d.	NB4, U937 and HL60 cell lines	Terminal: apoptosis induction	PERK/eIF2α-CHOP (C/EPB homologous protein)	[251,252]
PABA/NO	O2-[2,4-dinitro-5-(N-methyl-N-4-carboxyphenylamino)phenyl]1-(N,N-dimethylamino)diazen-1-ium-1,2-diolate	-	PDI	HL60 cell line	Terminal: Cytotoxic UPR	CHOP (C/EPB homologous protein)	[253]
alkyl gallate and gallamide derivatives		-	n.d.	HL60 cell line	Terminal: apoptosis induction	not well documented	[254]
Trichosanthin	type I ribosome-inactivating protein	596174	Ribosomes	HL60 cell line	Terminal: apoptosis induction	CHOP (C/EPB homologous protein)	[255]
auraptene	monoterpene coumarin ether	1550607	n.d.	Jurkat cell line	Terminal: apoptosis induction	Caspase 8	[256]
4-hydroxybenzylretinone	fenretinide analogue/synthetic retinoid derivative (related to vitamin A)	-	n.d.	HL60 cell line	Terminal: Cytotoxic UPR	CHOP (C/EPB homologous protein)	[257]
tipifarnib combined with bortezomib	quinolone and boronic acid	159324/387447	Farnesyltransferase Inhibiteur and 26 s proteasome inhibitor	KG-1, and U937 cell lines	Terminal: Cytotoxic UPR	not well documented	[258]
AEBSF	4-(2-aminoethyl) benzenesulfonyl fluoride hydrochloride	186136	serine protease inhibitor	NB4 cel line	Terminal: Cytotoxic UPR	not well documented	[259]
Thapsigargin (UPR inducer)	sesquiterpene lactone	446378	sarco/endoplasmic reticulum Ca++ ATPase	K562 cell line	Terminal: apoptosis induction	not well documented	[260,261]
arsenic trioxide (ATO) + kinase inhibitor imatinib mesylate (STI571)		14888/5291	BCR-ABL tyrosine kinase	K562 cell line and CML primary leukemic cells	Terminal: apoptosis induction	not well documented	[262]
Tetrocarcin-A		54681516	n.d.	CLL/T-ALL	Terminal: apoptosis induction	not well documented	[263,264]
